# Better Guidance Is Welcome, but without Blinders

**DOI:** 10.1371/journal.pmed.1001188

**Published:** 2012-03-20

**Authors:** David H. Peters, Sara Bennett

**Affiliations:** Johns Hopkins University Bloomberg School of Public Health, Baltimore, Maryland, United States of America

## Abstract

David Peters and Sara Bennett provide a critical perspective on a three-part series on health systems guidance that examines how evidence should be used to strengthen health systems and improve the delivery of global health interventions.

**Linked Policy Forums**

This Perspective discusses the following new Policy Forums published in *PLoS Medicine*:

Bosch-Capblanch X, Lavis JN, Lewin S, Atun R, Røttingen JA, et al. (2012) Guidance for Evidence-Informed Decisions about Health Systems: Rationale for and Challenges of Guidance Development. PLoS Med 9: e1001185. doi:10.1371/journal.pmed.1001185.

In the first paper in a three-part series on health systems guidance, Xavier Bosch-Capblanch and colleagues examine how guidance is currently formulated in low- and middle-income countries, and the challenges to developing such guidance.

Lavis JN, Røttingen JA, Bosch-Capblanch X, Atun R, El-Jardali F, et al. (2012) Guidance for Evidence-Informed Policies about Health Systems: Linking Guidance Development to Policy Development. PLoS Med 9: e1001186. doi:10.1371/journal.pmed.1001186.

In the second paper in a three-part series on health systems guidance, John Lavis and colleagues explore the challenge of linking guidance development and policy development at global and national levels.

Lewin S, Bosch-Capblanch X, Oliver S, Akl EA, Vist GE, et al. (2012) Guidance for Evidence-Informed Policies about Health Systems: Assessing How Much Confidence to Place in the Research Evidence. PLoS Med 9: e1001187. doi:10.1371/journal.pmed.1001187.

In the third paper in a three-part series on health systems guidance, Simon Lewin and colleagues explore the challenge of assessing how much confidence to place in evidence on health systems interventions.

The three-paper series on guidance for evidence-informed decisions about health systems, published in *PLoS Medicine*, and produced by members of the World Health Organization (WHO) Task Force on Developing Health Systems Guidance, offers important contributions to improving the quality of evidence-informed decision-making in health systems [1]–[3]. We recognize the importance of engendering greater structure and systematization in processes that collate and evaluate evidence, and bring it to bear on policy. However, there are significant challenges in doing this for policies related to health systems, and we caution against the adoption of rigid approaches to the development of guidance and to the application of evidence to policy.

In recognizing the growing international consensus on the importance of strengthening health systems, particularly in low- and middle-income-countries (LMICs), the first paper argues that better guidance is needed to provide evidence-informed decisions about interventions in health systems, analogous to the methods that have been used to develop clinical guidelines, and facilitate their implementation [1]. The second paper seeks to identify a series of practical processes and tools for policy development at international and national levels, and for developing guidance at the national level [2]. Many of the same authors have developed the Supporting Policy Relevant Trials (SUPPORT) tools [6] that provide a basis for a very systematic approach to organizing questions about health systems problems and decisions influenced by evidence (Tables 1–3 in [2]). The third paper attempts to adapt guidelines used in clinical evidence-based medicine to understand the quality of health systems evidence using the Grading of Recommendations Assessment, Development and Evaluation (GRADE) criteria [3],[7].

In the first paper, Xavier Bosch-Capblanch et al. identify multiple uses of guidance on health systems from a review of national policies and plans in LMICs, but for some of the guidance identified—such as the operational guidelines for procurement, human resource management, or planning and budgeting procedures—one wonders whether research evidence is critical. The authors offer little guidance as to where health systems guidance is most needed. Given the fairly resource-intensive approach proposed to producing health systems guidance a clear sense of priorities is required, and a recognition that sometimes adherence to “best practice” may be sufficient.

The papers pay relatively little attention to the well known “policy-implementation gap”, and sometimes appear to presume that getting policy right is sufficient. The WHO essential medicines program has encountered significant success in promoting the widespread adoption of an extensive set of guidelines (at global, national, and local levels) related to the development of evidence-based policies, institutions, and procedures [4]. Over 150 countries have adopted essential medicines lists based on thoughtful and evidence-informed guidance [5]. Yet, despite this significant success, policy implementation of essential medicines programs continues to face enormous challenges, with widespread irrational medicines use and a growing threat of counterfeit medicines. In practice, policy development is rarely a one-time event, but is rather a continuous process. National-level policy decisions may provide the overarching framework for change, but commonly the details of policy change are worked out on the ground through implementation processes and reflected in more informal expressions of policy such as ministerial memos and training manuals. While the second paper [2] in the series, by John Lavis et al., portrays a relatively clean process of interaction between global guidance and national guidance and policy, in practice there are likely to be multiple policy iterations as problems and issues merge. Accordingly, while establishing structured processes to promote evidence use, we must not lose sight of the importance of building networks of researchers and policy makers to facilitate ongoing dynamic interaction.

The authors of the third paper, Simon Lewin et al., note that systematic evidence is needed to address questions of feasibility and acceptability of interventions, as well as effectiveness, though much of the discussion in the paper addresses evidence regarding “what can work”, a question for which GRADE criteria function well. But policy makers may be more interested in questions such as “what can work in our (non-research) environment?”, “how can we make an intervention work well?”, or “how can we overcome obstacles to implementation in our situation?” They are also likely to be more concerned about the broader type of unintended consequences of an intervention (e.g., the political ramifications) [8], the type of results that are often not well examined by typical research on “what can work”. These alternative questions of interest to policy makers require different types of evidence than those that fit GRADE criteria. In addition, policy makers may have a different view about whether the type of inference they need to make is the same as those that drive scientists' questions on intervention effectiveness. They are unlikely to want to bind policy decisions to the ironically unscientific conventions of the health sciences with respect to probabilities of error, particularly when such *p*-values have little relevance to the nature of the policy question involved [9].

One implication of the realization that health systems, like most social systems, are actually complex adaptive systems, means that change often follows counter-intuitive and more complex patterns than is modeled through epidemiologic research on effectiveness embodied in the GRADE approach to evidence [10]. For example, the claim that a large effect size implies higher quality of evidence is likely to be true if health systems are deterministic or operate in a state of relative stochastic equilibrium, though this is often not the case in reality. In complex systems, small stimuli can lead to large effect sizes, and large interventions can lead to small change, but not in a very predictable way, particularly if the underlying phenomena are not well understood. Phenomena such as path dependency, emergent properties, and other non-linear patterns that occur in complex systems are often unmeasured in studies assessed to be high quality according to GRADE criteria, and thus such studies can lead to inappropriate inferences based on studies designed to fit GRADE criteria. Different scientific models may be necessary to interpret the quality of studies of complex systems [10]. An important practical implication is that a misplaced belief in simplistic systems can lead to poor policy decisions, frequently with policies that protect against small or moderate risks, but not against large-scale failure [11].

Although the papers together acknowledge that guidance needs to take account of contextual differences, the fragmented approach proposed to assess evidence on (i) effects, (ii) stakeholder views, and (iii) implementation issues suggests that the implications of context may not have been fully appreciated. From a systems perspective, stakeholder views, for example, matter in terms of how the population or other actors are likely to receive a health systems intervention proposal, and also fundamentally influence how that reform is implemented and the effects it creates. Actor resistance may lead to emergent behavior (such as seeking ways to “game” a provider payment system) that will in turn influence effectiveness. A better definition of health systems interventions than that proposed in this series would highlight the need for systems sciences and interdisciplinary inquiry and further expand the conceptualization of what comprises evidence.

Finally, one key assumption behind the approach to guidance development proposed is that systematic reviews provide the best type of evidence on the effects of policy options, but this is contestable. In the first place, one could argue that the best evidence is that which is experienced, learned, and acted on by key stakeholders in their own setting. While there is very little evidence about how policy makers in LMICs understand systematic reviews, based on our personal experience, we suspect that such understanding is often quite limited, and literature instead consistently points to the importance of personal interactions with researchers and locally produced evidence in the minds of policy makers. Systematic reviews clearly have an important place in the consideration of evidence, but as previously noted, there are many types of questions about policy options that are not well addressed by systematic reviews of effects or GRADE criteria that are weighted towards simple effectiveness studies. As the authors imply, methods for alternative types of reviews are still under development and continue to be debated and to some extent contested. Finally, although it is an important scientific principle embodied in the GRADE criteria that experiments need to be repeated to gain confidence in the validity of their findings, we also need to be cautious of the “fallacy of misplaced concreteness”, and particularly the assumption that because actions have been successful in some contexts, they need be under all conditions [8],[12].

The articles in this series point to a large agenda to better develop guidance to incorporate the different types of evidence needed for interventions in health systems, and have made a considerable contribution toward that end. Recognizing the diversity of stakeholders and complexity of health systems issues, it will be important to ensure that evidence-informed guidelines that emerge are tested with continued humility and skepticism, and that they do not become rigid models for inquiry dominated by a limited number of disciplines. They should not serve to blind us toward the need to address a wide variety of questions and incorporate the different types of evidence brought to bear by many fields of science. Further guidance is one important way to shape policy, but we must not fail to situate it in the broader context of sustained dialogue between researchers and policy makers.

## Abbreviations

GRADE: Grading of Recommendations Assessment, Development and Evaluation
LMIC: low- and middle-income-country
WHO: World Health Organization
